# Describing the implementation of an innovative intervention and evaluating its effectiveness in increasing research capacity of advanced clinical nurses: using the consolidated framework for implementation research

**DOI:** 10.1186/s12912-017-0214-6

**Published:** 2017-05-02

**Authors:** Gabrielle McKee, Margaret Codd, Orla Dempsey, Paul Gallagher, Catherine Comiskey

**Affiliations:** 1 School of Nursing and Midwifery, Trinity College Dublin, 24 D’Olier St, Dublin, Ireland; 2 St James Hospital , Dublin, Ireland; 3 Post-Doctoral Fellow and Quantitative Healthcare Lead, Centre for Practice and Healthcare and Innovation, School of Nursing and Midwifery, Trinity College Dublin, Dublin, Ireland

**Keywords:** Research capacity building, Clinician researcher, Nurse role, Nursing research, Programme evaluation, Research participation

## Abstract

**Background:**

Despite advanced nursing roles having a research competency, participation in research is low. There are many barriers to participation in research and few interventions have been developed to address these. This paper aims to describe the implementation of an intervention to increase research participation in advanced clinical nursing roles and evaluate its effectiveness.

**Methods:**

The implementation of the intervention was carried out within one hospital site. The evaluation utilised a mixed methods design and a implementation science framework. All staff in advanced nursing roles were invited to take part, all those who were interested and had a project in mind could volunteer to participate in the intervention. The intervention consisted of the development of small research groups working on projects developed by the nurse participant/s and supported by an academic and a research fellow. The main evaluation was through focus groups. Output was analysed using thematic analysis. In addition, a survey questionnaire was circulated to all participants to ascertain their self-reported research skills before and after the intervention. The results of the survey were analysed using descriptive statistics. Finally an inventory of research outputs was collated.

**Results:**

In the first year, twelve new clinical nurse-led research projects were conducted and reported in six peer reviewed papers, two non-peer reviewed papers and 20 conference presentations. The main strengths of the intervention were its promptness to complete research, to publish and to showcase clinical innovations. Main barriers identified were time, appropriate support from academics and from peers. The majority of participants had increased experience at scientific writing and data analysis.

**Conclusion:**

This study shows that an intervention, with minor financial resources; a top down approach; support of a hands on research fellow; peer collaboration with academics; strong clinical ownership by the clinical nurse researcher; experiential learning opportunities; focused and with needs based educational sessions, is an intervention that can both increase research outputs and capacity of clinically based nurses. Interventions to further enhance nursing research and their evaluation are crucial if we are to address the deficit of nurse-led patient-centred research in the literature.

**Electronic supplementary material:**

The online version of this article (doi:10.1186/s12912-017-0214-6) contains supplementary material, which is available to authorized users.

## Background

Across the world there has been significant expansion of the nursing role and the competencies within it. These have already demonstrated added value to patients and services [1, 2]. Within the more advanced nursing roles one of the competencies includes research. With the expectancy of advanced nursing staff to be research active and undertake research there is clearly a need not only to provide research capacity development and ongoing support but also to evaluate it’s effectiveness. The impact of these roles on patient care is already evident. This may in part be due to the increased research role, enabling quality improvements to be even more systematic and more evidence based [3]. There is a strong need in the literature for more research that is patient centred [4]. These new roles and their impact on practice can provide this. But it can only do this if there is sufficient research capacity within the nurses in these roles.

Research capacity building is a broad concept that encompasses some or many aspects of research ranging from awareness, knowledge, skills, understanding, utilisation, data collection, presentations through to participation [5–7]. While there is an identified inconsistency in the meaning of the term research capacity building, within this paper it refers to involvement at all levels of the research process from question design to dissemination [5, 8]. Research capacity building has been identified as a priority in nursing research and development [5, 9]. However, within nursing to date the emphasis in research capacity building has been mainly in nurse academics [5, 9, 10], rather than in nurses with a clinical role [7, 9, 11, 12].

Clinical nurse participation in research is relatively low [7, 11]. Many barriers to participation have been identified. Personal barriers include nurses’ attitudes [13, 14], poor knowledge about research [7, 12–16], lack of opportunity or experience [14, 15, 17], need for research skills [9, 14, 17, 18], lack of qualifications [12], lack of interest [7], lack of confidence [14, 15] and lack of motivation [13–15, 19]. At organisational level, other barriers to research have been identified and include, lack of research supervision [7], lack of support [7, 11, 13–17, 20], lack of reward [11], power hierarchies [12, 14], lack of mentorship [12], lack of time [7, 11–13, 15, 17, 18, 20, 21], and poor resources and funding [11–16, 18, 22].

Review of the literature indicates that initiatives to enhance clinical research capacity should address three main areas; leadership, expertise and capacity [11, 23]. Strong leadership has long been advocated as a key element in clinical research capacity building [8, 10, 21, 22, 24]. Other features that reflect a strong leadership presence include, the development of a strategy, formalising research policies and support [8, 20, 24, 25], identifying priorities [24], the development of a research culture [15, 20, 22], an organisational need for research [8] and the use of a steering committee [11, 20].

The features needed to provide expertise and capacity have some common characteristics. The use of collaborations or networks [8, 22, 23, 26, 27], is a common feature of support advocated [25]. This can be of several different forms but usually embraces using experts in the area [8], either academics [11, 28] or other clinicians both within and outside of the discipline [13] and utilising different modes of mentorship [29].

Sourcing funding is also strongly advocated as a method of providing support. Whether this is to provide direct support to single researchers, finding funding for research facilitator posts or other resources [8, 25], investments in infrastructure or other aspects of an initiative [20, 25] or building elements of sustainability and continuity [11]. Support can also be provided through the development of an educational programme or educational providing opportunities [8, 9, 13, 19, 23, 25, 30] or through the use of journal clubs [13] newsletters and monthly research meetings [19]. But overall it is evident that the leadership model utilised should provide a good support management system that will, in addition to the above, increase awareness of research within units [8, 25] so as to increase the capacity of individuals to engage in research.

Additional features that have been advocated in interventions used to enhance clinical research capacity building include ensuring the research is ‘close’ to practice, that the clinical staff identify the research ideas [11] or jointly identify the ideas [28], that small research teams are used [19, 20, 31], that these are clinician led [20, 31] and that the outcome of the research not only informs local practice but is disseminated [8, 11, 25, 28, 30].

Paget et al. [18] concluded that while interventions to increase capacity in nurses are well received the evidence of their effectiveness is limited. This is well evidenced from the paucity of studies evaluating the effectiveness of research capacity building initiatives [4, 11, 12, 27, 31, 32]. A further barrier to the measurement and dissemination of the effectiveness of interventions is the lack of use of consistent tools [33]. Despite these limitations, it has been shown that when some of the key aforementioned features are present there is an increase in knowledge post-intervention [13, 20, 32]. Post-intervention there was also a change in attitude towards research and a culture of research was developed resulting in an increase in research related activities [13, 25, 27, 31]. The evaluations identified many of the strengths of the implementations such as support, leadership, collaboration, visibility but also identified still outstanding weaknesses such as time and resources [26, 27, 31].

So although there has been a vast amount of literature on the barriers to research participation by clinical nursing staff, and a lot of literature about the elements that would promote good research capacity, much more is needed with regard to the evaluation of interventions implemented to improve research capacity.

It was evident from the literature that a successful standard intervention did not exist, but that local, national, policy and other influences have to be taken into account. Future interventions must not only evaluate their outcomes and effectiveness but contextualise them thoroughly. To address this, suitable frameworks need to be utilised in development, implementation and reporting of such interventions. The relatively recent developed consolidated framework for implementation science, as originally described by Damschroder et al. [34] and further detailed in Breimaier et al., [33–35] would be appropriate. This framework has already been utilised widely in research in general and in health sciences research [36]. It is a consolidation of many theories and therefore provides a comprehensive framework, uses standardised structures and common language that allow readers to identify the most important elements of all phases of an implementation. In particular, it allows for the contextualisation of the intervention to be systematically addressed, which as seen from the literature is essential for future research in this area.

The aim of this paper is to describe the implementation of an intervention and evaluate its effectiveness in increasing research capacity in advanced clinical nursing roles.

## Methods

### Research design and conceptual framework

The evaluation utilised a mixed methods design and the consolidated framework for implementation science as a conceptual framework.

### Intervention

From inception to analysis the intervention developed to increase research capacity in advanced clinical nursing in an acute urban hospital setting, utilised the consolidated framework for implementation science. This methods section describes the intervention using the five domains of this framework. These are, intervention characteristics, the social context, the local context, the characteristics of the individuals involved and the process including evaluation [34, 35].

#### Intervention characteristics

**Fig. 1:**
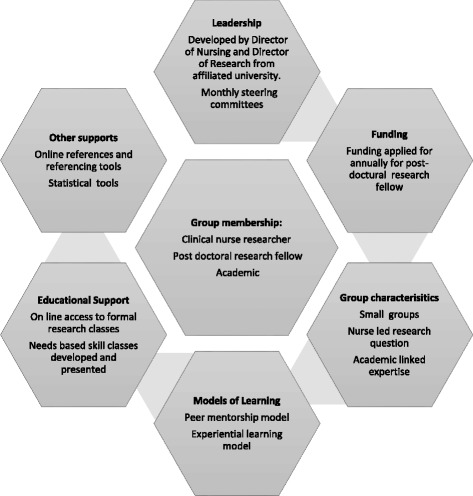
Outline of intervention components

The intervention developed included, skill development, support with quantitative methodology and support from researchers experienced in both carrying out research and research dissemination. The key characteristics of the intervention were leadership, steering group, funding, small research groups with post-doctoral research fellow support, clinical and academic nurse researchers, focused clinical nurse-led research questions, peer mentorship, experiential learning and, the provision of additional educational sessions based on needs (Fig. 1). Funding was sourced to employ a part time post-doctoral researcher with quantitative and health research experience to act as a research facilitator and provide both expertise and sharing of research workload with the nurse researchers.

Through the utilisation of the skills of both clinical and academic partners the intervention aimed to facilitate the understanding, development and application of research skills, to produce demonstrable outputs for patient care and research, e.g. statistical analysis, study design, academic writing and submission of papers for publication, and practice development.

#### Outer setting (Social setting)

The implementation of the intervention facilitated development, implementation, evaluation and dissemination of clinical innovations within the hospital, all of which were believed to have a direct effect on patient outcomes. To increase the quality of the implementation, in addition to the international literature evidence, the academic partner consulted with international clinical and academic institutes who had interventions in place for increasing research in clinical nursing staff.

#### Local context (Inner setting)

The hospital site was a large (1020 bed) urban acute teaching hospital with several national disease centres and the academic partner was a university School of Nursing and Midwifery, both in Ireland. Nursing within the hospital had a well-developed centre for practice development that mentored and supported innovation and development in practice. However, there was no existing formal research infrastructure to support the research needs of the new advanced clinical nurses on a formal basis.

### Characteristics of participants

The development was driven by leaders high in the hierarchy of the hospital (Director of Nursing and Assistant Directors of Nursing) in collaboration with research personnel in the linked academic institute (Director of Research and the Director of the Centre for Practice and Healthcare Innovation). The steering group included the above (only one Assistant Director of Nursing), the Nurse Practice Development Co-ordinator and the Head of the Centre for Learning and Development.

The inclusion criteria, for participation in the intervention, were nursing staff at clinical nurse specialist or advanced nurse practitioner level (advanced nursing roles) with a clinically based research topic of interest they wished to develop or wanted support with. Research nurses were not eligible to apply. Peer mentoring was to be provided by the academics within small research groups.

### Process

The intervention’s development and implementation took place from 2010 to 2013. The planning, engaging the participants and executing of the interv﻿ention are described herein.

Based on the literature an intervention with the aforementioned elements was developed by the steering group (Fig. 1). Table 1 indicates the main steps in the development of the innovation, the purpose, rationale, actions and the main stakeholders involved.

**Table 1:** Key steps in the development of the intervention

Phase	Feature and timeline	Purpose	Action	Responsibility/who involved
Planning	Steering group: Commenced April 2010	Planning and development.	Develop intervention Alert staff to intervention development Call for interested staff to partake in intervention.	Members of the steering group
	Funding : First funding received September 2010, funding applied for on a biannual basis	Source funding for post-doctoral research fellow.	Funding sourced for part time post-doctoral research fellow	Members of the steering group
	Set up small research groups : First groups set up September 2010		Small research groups established	Steering group/members of research groups
Implementation	Steering group	Provide leadership and support and increased awareness of change in role of staff and supports available.	Monthly review meetings of progress of teams, information about initiative relayed to all levels of nursing management and academics, encouragement and motivation from senior nursing staff to nurse researchers.	Members of the steering group
	Small research groups	Support, peer collaboration between academics and nurse researchers and experiential learning.	Conducted research.	Post-doctoral research fellow/clinical researchers/academic researchers.
	Post-doctoral researcher:	Support research teams, particularly through collation and analysis of data.	Arranged meetings, conducted aspects of the research.	Post-doctoral research fellow
	Research skills classes	Increase research capacity.	Developed or facilitated by nursing and academic members of the steering group, classes provided both face to face and online.	Steering group members.
Evaluation	Audit of research metrics: December 2015	Audit of dissemination/research output.	Audit presentations and publications. Team leaders provided information to research facilitator on a monthly basis.	Research group leaders
	Survey of skill utilisation and development: March 2012	Evaluation of changes in research capacity.	Development of questionnaire, data collections and analyses.	Research group members
	Focus groups: May 2012	Evaluation of effectiveness of the intervention and barriers to its optimisation.	Focus groups.	Research group members

The Director of Nursing mailed all the advanced clinical nurses in the hospital and invited those with a research topic they wished to develop or have support with, to submit an outline of their project to the steering committee. Academics with relevant experience in the research topic or methodology of the advanced nurse projects were invited to become members of the small research groups. Groups therefore included the advanced clinical nurse/s who had developed the research question, the postdoctoral research fellow and usually one academic.

The small research groups then developed their own schedule of meetings, work and appropriate target outputs. The different aspects of the research process ranging from searching the literature to research dissemination were carried out by any appropriate member of the group, achieving a balance between utilising research strengths and developing areas of weakness. The data analysis was mainly the role of the post-doctoral research facilitator and the data cleaning was carried out by both the post-doctoral research facilitator and the advanced clinical nurse researcher. It was planned that within the research groups there would be experiential development of research skills using peer mentorship in a supportive, collaborative team environment.

To further enhance the research skills of the clinical nurses additional direct support mechanisms were introduced. The clinical nurse researchers were invited to attend the research support seminar and methodological master classes that ran in the university nursing school on a monthly basis. These sessions were also made available online so that the clinical nurse researchers could access them at a time that was convenient to them. In addition to this, an annual one day series of workshops and seminars was conducted in order to build on and develop research skills. Based on feedback these talks centred on research dissemination and included talks on how to write an abstract, writing etc. Following the establishment of the research groups the steering group took on the role of monthly monitoring of progress and offering additional support and guidance to the groups and to the post-doctoral researcher in their role as facilitator.

### Data collection

There were two minor quantitative measures. Firstly, a survey, with a self-reported research skill utilisation and development questionnaire. This was developed by two members of the steering group. Content and face validity were conducted, it was then piloted by the remainder of the group and an additional three academics. All comments were reviewed, questions rephrased deleted or inserted and reviewed again. The final version surveyed the three main areas in the research process, broken down into 24 separate skills, including, searching and critiquing literature, data collection, cleaning and input and writing and dissemination skills. The nurse researchers reported which aspects of research they were involved in during the intervention and also ranked their perceived skill level/experience pre- and post- intervention implementation on a five point Likert scale from 1: no experience or education in this area, to 5: have significant experience in this area (Additional file 1: Research skills and activities questionnaire).

The second quantitative measure was an audit of the research outputs arising from the intervention i.e. publications and disseminations (Additional file 1: Research skills and activities questionnaire).

The major evaluation element was qualitative. It consisted of two focus groups assessing the barriers and benefits of the intervention.

Questionnaires were disseminated to participants by email to those who fulfilled the inclusion criteria approximately 18 Months after implementation of the intervention. The mail content acted as the participant information leaflet and consent was inferred if the participant choose to complete and return the questionnaire. Participants were given the choice to return the questionnaire by email or anonymous copy. The aim of the survey was to give a crude indicator of the aspects of the research process the participants were involved in within the intervention and to assess skill change pre- and post-intervention.

Two focus groups were conducted at around 20 months following implementation of the intervention. The first focus group inclusion criteria were nurse researchers and academics partners who partook in the intervention. The potential participants were emailed inviting them to the focus group. The mail content acted as the participant information leaflet, informing the participant of the voluntary nature of participation, the objectives of the focus group, the manual recording of the discussion, the confidentiality of the process and the fact that the content of the discussion would be disseminated. In a similar manner, members of the steering group were invited to attend a second focus group. The focus groups were facilitated by an experienced facilitator who was a member of the steering committee with the assistance of a note-taker. Consent was taken at the beginning of the focus group. To assist in protecting confidentiality the names of attendees or names mentioned in the focus groups were not recorded. A Strength-Weakness-Opportunity-Threat (SWOT) approach was adopted to guide the conduct of both focus groups. After each focus group notes were written up for analysis.

Finally, the participants of intervention, reported to the steering group detailed information pertaining to their successful outputs. The outputs were then collated and summarised. The outputs presented here are those completed up to December 2015.

### Data analysis

The questionnaire data were collated, then anonymised and analysed using SPSS (v21). They were analysed at the descriptive level using frequencies and percentages. Members of the steering committee read and examined the findings to ascertain the quality of the research [37].

A deductive approach to the analysis of the qualitative data was taken [38]. Thematic content analysis was carried out using a pre directed or theoretically driven format [39]. Data from the focus groups were analysed using a SWOT analysis framework. Data were manually coded into strengths, weaknesses, opportunities and threats and subsequently into themes and subthemes. The resulting analysis framework was checked by facilitators to ensure validity of the findings. The analysis was conducted within a realistic perspective which aimed to report experiences, meanings and the reality of the participants [39].

## Results

In line with the first inclusion criteria all nursing staff in advanced nursing roles were invited to partake in the intervention (*n* = 72). In line with the second aspect of the inclusion criteria, these invitations asked those with a clinically based research topic of interest they wished to develop or like support with, to volunteer to be part of the intervention. A total of 17 advanced nurses met the eligibility criteria and responded to the initial call. In the first year 12 clinical nurse researchers participated in the intervention. These nurses were all at an advanced level and therefore had qualifications up to masters’ level.

The research groups emerged across many different disciplines within the hospital and included projects in the following fields: haemophilia, tracheostomy safety, Human Immunodeficiency Virus (HIV) and oncology. It covered areas such as; developing research questions and data collection tools; completing ethics applications; collecting data; developing research questions for data already collected; bringing research previously undertaken to publication; developing and evaluating innovations and implementing and evaluating guidelines. Projects took on average up to two years to complete. Since 2011, an additional two to three new projects are undertaken each year. Currently there are 15 active projects being undertaken.

### Self-reported research skill utilisation and development

Seven of the 12 clinical nurse researches from the groups completed evaluation questionnaires, a response rate of 58%. Self-reported research skill level was relativity high (>3 on a scale of 1-5), prior to the intervention in most areas examined, with the exception of data cleaning and writing the background for a paper (Table 2). The most common research activities the clinical nurse researchers participated in during the course of the intervention were writing conference abstracts (*n* = 6, 86%) and reviewing drafts of their projects’ paper (*n* = 5, 71%) (Table 2). The greatest skill change occurred in data cleaning. Moderate skill changes were seen in, setting up databases, cleaning data, data analysis, writing background of paper, writing methodology, writing discussion, creating tables and presenting at an external conference (Table 2).

**Table 2:** Participants self-reported involvement in the research process pre and post intervention

Role in the research project	Number	Percent	Mean perceived skill level	
			Pre-project	Post-project
Searching literature	4	57	3.50	3.50
Critiquing literature	4	57	3.50	3.50
Writing the background literature	4	57	3.25	3.75
Developing the research question	4	57	3.00	3.50
Sourcing data collection tool	4	57	3.75	4.00
Developing data collection tool	3	43	4.00	4.33
Data collection	4	57	3.50	3.75
Data cleaning	2	29	2.50	4.00
Setting up database	1	14	3.00	4.00
Cleaned data	1	14	3.00	4.00
Data analysis	2	29	3.00	4.00
Data interpretation	4	57	3.50	4.25
Writing background of paper	2	29	2.50	3.50
Writing methodology	3	43	3.00	4.00
Writing discussion	1	14	3.00	4.00
Writing report	3	43	4.00	4.67
Writing abstract	6	86	3.83	4.33
Reviewing drafts of paper	5	71	3.20	4.20
Creating tables	1	14	3.00	4.00
Creating diagrams	1	14	4.00	4.00
Preparing poster	4	57	3.50	4.25
Preparing PowerPoint presentation	2	29	4.50	4.50
Presenting at an internal conference	3	43	4.33	4.33
Presenting at an external conference	2	29	3.00	4.00

### Focus group findings

There were five clinical nurse researcher attendees and two academics at the first focus group and six attendees at the steering group focus group. The emerging themes from the two focus groups were drawn together under two overarching themes: 1) strengths and opportunities and 2) weaknesses and threats.

#### Theme 1: Strengths and opportunities

Within this theme two sub-themes emerged – partnership and outcomes.

##### Subtheme 1a Partnership

This was a strength identified by both focus groups. The main categories that emerged under this subtheme were teamwork, support, collaboration and goal setting (Table 3). Participants felt teamwork was productive and comfortable and it provided support for all participants, in particular there was praise for the support of the post-doctoral research fellow.

“*Couldn’t have done it on my own*”

**Table 3:** Focus group findings

Themes	Subthemes	Categories	Codes
Strengths and opportunities	Teamwork	Teamwork	Productive and comfortable Good relationships Team approach Experience of working with a team Motivational Couldn’t do it on my own
		Support	Support from post doctural researcher Support for all
		Collaboration	Opportunity to collaborate in new ways as partners Clear collaboration Merging of expertise
		Goal setting	Deadlines incentivised work
	Outcomes	Publications	Multiple dissemination methods Diverse range of projects Enhancing hospital’s research image Showcase hospital’s good practice
		Skill development	Learning about methodology Increased skills Time management skills Working a variety of skills More onsite skill development Learned how to select appropriate journal Confidence
		Role development	Enhanced role Fulfil research role
		Impact	Practice development Bringing it back to practice Model of use elsewhere Publish completed research Development of innovative research centre Promotes initiative to develop clinical professor on nursing
Weaknesses and threats	Time	Time	Time to conduct research Time for the meetings Time for correspondence Research time not protected Takes a lot of time Put under pressure to complete in time
	Awareness	Awareness	Lack of awareness about how much time the different aspects of research takes (data cleaning, publication) Intervention needs to be to be promoted more Lack of awareness of those not involved in the projects
		Value	Seen not to be pulling your weight in practice if doing research Voluntary aspect of participation
	Concerns	Issues	Access to resources such as SPSS Skill match not always optimal Frustration at not getting published Lack of consistent methods of recording things made data extraction too difficult Capacity of post doctural researcher challenged to meet needs Need to get ethics approval Worries about ownership of data Funding to maintain intervention
		Missed opportunities and Unmet needs	Involve more groups Keen to develop more research skills, quantitative, action research, writing skills

A further strength of the intervention was the opportunity to collaborate in new ways, as partners, merging expertise to enable the practice research to be completed and showcased. Deadlines set by the project groups themselves emerged as a strength that incentivised work and pushed group members to achieve deadlines for their research tasks within an already busy schedule.

##### Subtheme 1b Outcomes

Under the outcomes subtheme several categories emerged, namely, publications, skill development, role development, and impact. The number of publications and the multiple dissemination methods used to promote the nursing research profile of the hospital site were identified. Several participants commented on how the intervention offered the stimulus, motivation, opportunity and support to publish findings from previously completed and unpublished research untaken as part of a Master’s degree.

*“Means/forum to get the research findings out there”*

Participants also highlighted how the innovation facilitated the opportunity to work and develop a variety of research skills. These included methodology knowledge and utilisation, writing and reviewing work, the submission process and developing further research questions. They also reported developing their time management skills.

Clinical impact emerged as an outcome of the innovation. Participants expressed how their involvement in the innovation increased their observation of their own clinical practice, brought the research back to practice, enhanced practice development and the clinical role overall while contributing to improved patient care.

*“Not just writing around (theory) – kept bringing it back to practice”*

*“Brought the innovations (practice development) to another level”*

Participants also noted that the innovation enhanced the nursing research image both within the hospital and externally.

#### Theme 2: Weaknesses and threats

Several subthemes emerged from this theme. These were time, awareness of what research involved and general concerns (Table 3).

##### Subtheme 2a Time

Time was a significant barrier to participation in the innovation and this was cited by both the steering committee and the researchers. Specific aspects with regard to time included, making time to do the research, making time for meetings and lack of protected time for research.

*“Put under pressure, when no protected time at work”*

This was further compounded by the lack of awareness with regard to the amount of time research takes, particularly the unseen elements such as cleaning data, ethics submission process and ﻿article submission and resubmission ﻿process.﻿

##### Subtheme 2b Awareness

An important aspect of the innovation that arose from the focus groups was the need to increase awareness across the hospital of staff involvement in research and recognition that this was part of their work.

*“Colleagues felt I was removed, spent a lot of time on it* (research)*”*

The voluntary participation of academic nurse researchers or clinical nurse researchers was seen as a weakness. It also emerged that the value of the intervention in the long term would be threatened if workload, equality and awareness were not addressed within the hospital.

##### Subtheme 2c General concerns

Academics were matched to projects from either a topic of interest or a methodology expertise point of view but not always both. This was cited as a weakness. Related to this some teamwork was not always optimal, and in some cases lack of clarity of dates, roles and tasks led to frustration and loss of time.

*“Wasn’t always clear of the submission date* (to team)*”*

Awareness of the ethical issues of research including ownership also emerged as threats. Data extraction from retrospective data proved more time consuming than expected and in some instances valuable data was available but the original data collection methods made data extraction impossible from a time point of view. Lack of access to resources like statistical packages within the wards of the hospital proved frustrating and contributed to the missed opportunity for further statistical and analytical skill development of the clinical nurse researchers. In addition, participants experienced frustration, worry and fear that they may be conducting research which may not be published.

### Audit of research output metrics

These outputs included six peer reviewed papers, two non-peer reviewed papers, ten international conference presentations, seven national conference presentations and three local conference presentations (Table 4).

**Table 4:** List of main dissemination outcomes from the twelve initial projects

	Project Topic	Dissemination	Format
1	Advanced role in colorectal screening	[41]	Publication: Non-peer reviewed, national, generic nursing journal
2	Achieving Impact through Human Factors Research	[42]	Publication: Peer reviewed, national, topic specific journal
3	Nursing Care of Older Persons with Dementia		Presentation: National, generic nursing conference
4	Reperfusion times in patients with AMI in emergency department		Presentation: National, topic area conference
5	Development of Haematology Oncology Telephone Triage System		Presentation: International, topic specific conference Presentation: National, topic specific conference Presentation: Local, multidisciplinary conference Presentation: Local, multidisciplinary conference
6	Evaluation of the effectiveness and cost of a nurse-led risk assessment tool to reduce the incidence of febrile neutropenia in adult cancer patients receiving chemotherapy	[43]	Publication: Peer reviewed, disease specific nursing journal Presentation: International, generic nursing conference Presentation: International, generic nursing conference Presentation: National, generic nursing conference Presentation: National, topic specific conference
7	Nurses’ Knowledge and Standards of Tracheostomy Care Since the	[44]	Publication: Peer reviewed, topic specific journal Presentation: International, medical conference, published Presentation: International, generic nursing conference Presentation: International, topic based conference
8	Patient controlled analgesia	None	None
9	An evaluation of patient outcomes following Transcatheter Aortic Valve Implantation (TAVI)	[45, 46]	Publication: Non-peer reviewed, national, generic nursing journal Presentation: International, generic nursing conference
10	Development of a model of quality evaluation and improvement within a haemophilia service: moving from patient involvement through patient participation to patient partnership.	[46]	Publication: Peer reviewed, disease specific journal Presentation: International, nursing topic conference Presentation: International, disease specific topic conference Presentation: International, healthcare conference Presentation: National, disease specific conference
11	Client satisfaction with a nurse led carrier testing clinic and counselling service	[47]	Publication: Peer reviewed, generic nursing journal Presentation: Local, generic nursing conference
12	Living with HIV and Hepatitis C Virus	[48]	Publication: Peer reviewed disease specific nursing journal Presentation: National, generic nursing conference

## Discussion

The social context of advanced nursing in Ireland and internationally dictated a change in research involvement for newly created and growing advanced nursing roles. However, within the local context, the fact that a research culture was not embedded in the site did cause issues in the implementation of this change as seen in previous literature [8]. This intervention was put in place to address some of these issues by developing a supportive nurse-led research intervention. This intervention proved successful in increasing research capacity and research output of clinical nurses with a research competency attributed to their role.

The intervention developed was a mixed internal and external solution to an identified need of more support for research for advanced clinical nurses with research as part of their role.

The intervention is adaptable and was tailored to the resources and needs of the hospital. The design of the intervention is evidenced based, informed by both the known barriers to research participation by clinical staff and the facilitators to research participation. As indicated in the background there was previous evidence of the strength and quality of some of the different elements within the intervention but an additional strength was the collective use of several of these elements. One of the key strengths of this intervention as recognised in the evaluation and highlighted in previous interventions, was the ongoing support of the Director of Nursing and leadership provided by the steering committee who met monthly [8, 18]. The intervention facilitated the development of research expertise, it was close to practice, substantiated the linkages with the university, facilitated collaboration between practice and the university, provided access to educational opportunities and utilised already established resources.

This study supports the previous literature in the area, indicating that a partnership – peer mentorship model, that included a clinical nurse researcher in practice, a nurse or healthcare academic and a post-doctoral research facilitator supported by a high level steering group, improved research output [4, 8, 12, 20, 26, 30, 31]. An increase in research capacity was also demonstrated from both the quantitative survey of outputs and the focus groups. Supporting previous findings, the main benefits and strengths emerging from the intervention were its promptness to complete research, the chance to publish and showcase innovations and the opportunity to collaborate [8, 11, 25, 28, 30]. This was evident not only in the focus group findings but is also supported by the number, range and quality of the disseminations from the audit of publications.

However, several challenges and barriers remained. All of the participants had masters’ level qualifications and had previously partaken in short quantitative analysis courses and methodological classes were available within the intervention. Despite this, the questionnaire results indicated that participation in and the advancement of analytical skills was low. In addition within the qualitative findings, like other studies, nurses were still keen to receive even more input on skills such as statistical and analytical skill development [17]. The focus groups gave a clear message that time was a major issue, and elements such as data cleaning took a lot of time. Although it would be desirable that advanced clinical nurses were statistically well educated which would enhance the quality of the research experience and the outputs from the research, is it the best use of their time to engage in data cleaning etc.? The intervention developed here was similar to the research model adopted in many parts in academia where the roles are divided. For future development of the intervention we have to work out what is most time effective.

Resources are always an issue for nursing research [8–13]. This externally financed intervention reduced the time needed for research by providing statistical and analysis resources and academic support. The funding for the intervention is sourced like research funding. The post-doctoral researcher position is not funded from central hospital sources but has to be secured externally annually or biannually. This begs the question of the vulnerability of the intervention without this support and does it have enough elements of sustainability and continuity without it? It is envisaged that because of the collaborative links formed with the affiliated academic institute and the increased skills of the participants to date, that the intervention would be able to continue but to a limited extent. The drive and motivation that is offered via the link to the academics and post-doctoral researcher would be missed. Other advanced nurses who have not to date been involved in the intervention may lose out, the work involved in developing research would be a greater challenge and burden.

The proportion of nurses in advanced roles that had a research question ready and were able to partake in the intervention was low (17%), which needs to be explored further to see what individual or local context barriers remain an issue to participation in research. This is a weakness as it may prevent the development of a culture of research development within the site [13, 25, 27, 31]. Although the intervention facilitated the successful attainment of competencies there was no incentive except personal, for publication. The implementation did provide an ideal microclimate for learning and development. Of those who did participate, in support of previous literature, this study found allocating time for research was still an ongoing challenge [5, 8, 9, 11, 12, 18, 19]. It is still difficult for research to be seen as a priority given clinical commitments in spite of supports at senior level. Having protected time for research may help address this issue. While there was a perceived need for the implementation of this intervention, the timing of the implementation was far from perfect. It occurred at a time when there was an embargo within the hospital on staff recruitment so staff were stretched to fulfil the direct patient centred competencies of their role never mind their research and audit competencies, therefore these could not be prioritised.

Another contributing factor to poor uptake and previously documented as a barrier to research may be the lack of awareness of the intervention across the hospital [8, 25]. Increasing awareness and visibility of the intervention and the support from the senior ward staff should further assist in nurses spending time at research without causing animosity from colleagues. The time has come to further develop the intervention. This could be achieved by consideration of getting more permanent funding for the intervention, providing protected time for research and increasing the research role visibility as a non-optional aspect of the advanced clinical nurse role. The establishment of this intervention as a permanent feature will be threatened if funding, workload, equity and awareness are not addressed.

## Conclusions

While there are many barriers to optimising the research role of nursing staff with a research competency in their role, there are few studies that present, implement and examine the effectiveness of interventions to optimise this role. This study shows that a parsimonious intervention, with minor financial resources, that includes top down ongoing and committed support, support of a hands on research facilitator and peer collaboration with academics on projects that maintain strong ownership by the clinical nurse researcher, is an intervention that can both increase research outputs and research capacity of advanced clinical nursing staff. Further work is required to develop research policies and build elements of sustainability and continuity so that ongoing barriers to participation, such as protected time, are addressed. Interventions to further enhance nursing research and their evaluation are crucial if we are to address the deficit of nurse led patient-centred research in the literature.

### Limitations

This study is limited in several aspects. The sample size for the survey is small (*n* = 7, 58%) therefore, care needs to be taken in interpretation of the results. However, Hackshaw [40] indicates that well-designed small studies are acceptable once results are interpreted with care. Also the voluntary nature of participation means that there is the potential for non-response bias. The questionnaire was developed and tested by the research team and the item scores are somewhat subjective, further reliability testing would be required if the evaluation was to be repeated. The questionnaire was self–report which consequentially is a limitation. The steering committee, as key stakeholders in the implementation of the intervention, could be said to have an investment in positive evaluation results. Finally, due to the anonymous nature of the questionnaire it was not possible to integrate findings from the qualitative and quantitative aspects of the evaluation.

## Additional file

Additional file 1: Research skills and activities questionnaire. Questionnaire evaluating self-reported research activities and skill acquisition and changes in these pre and post intervention. (DOCX 19 kb)
