# Supplementary material for: Describing the implementation of an innovative intervention and evaluating its effectiveness in increasing research capacity of advanced clinical nurses: using the consolidated framework for implementation research
Source: BMC Nurs. 2017 May 2;16:21. doi: 10.1186/s12912-017-0214-6 (PMC5414169; doi:10.1186/s12912-017-0214-6)
Supplement: Additional file 1: — Research skills and activities questionnaire. Questionnaire evaluating self-reported research activities and skill acquisition and changes in these pre and post intervention. (DOCX 19 kb) [file 12912_2017_214_MOESM1_ESM.docx]

**Evaluation of St James Hospital and School of Nursing and Midwifery TCD Joint Nursing Research Initiative**

You are eligible to return this questionnaire if you were directly involved in this research innovation.

| **Section A** | | | |
| --- | --- | --- | --- |
| **Project Topic:** | | | |
| **Project team:** | | | |
| **In the first column tick all of the phases or roles you undertook during the last year** **in the collaborative projects you have been involved in i.e. contributed to more than 20% of the work in this aspect of the research project.**  **In the latter two columns rank your skill/education/ experience from 1-5 before and after this involvement:**  1 = no experience and no education in this area  2 = no experience but some education / a little experience but no education  3 =have a little experience and education in this area  4 = have some experience of this  5 = have significant experience of this | | | |
|  | **Was involved in this aspect** | **Perceived skill level /experiences before project** | **Perceived skill level /experiences after project** |
| Searched literature |  |  |  |
| Critiqued literature |  |  |  |
| Sourced a data collection tool |  |  |  |
| Developed research question |  |  |  |
| Developed data collection tool |  |  |  |
| Data Collection |  |  |  |
| Data input |  |  |  |
| Data cleaning |  |  |  |
| Set up of database |  |  |  |
| Cleaned data |  |  |  |
| Data analysis |  |  |  |
| Data interpretation |  |  |  |
| Writing background of paper |  |  |  |
| Writing methodology |  |  |  |
| Writing discussion |  |  |  |
| Writing of report |  |  |  |
| Writing an abstract |  |  |  |
| Reviewing drafts of paper |  |  |  |
| Creating tables |  |  |  |
| Creating diagrams |  |  |  |
| Preparing a poster |  |  |  |
| Preparing powerpoint research presentation |  |  |  |
| Presenting at an internal conference |  |  |  |
| Presenting at an external conference |  |  |  |
| **Section B**  **List of dissemination outcomes:** *Nominate a member of the team to complete this section to avoid duplication. Please cut and paste and duplicate the sections below if applicable.* | | | |
| 1. *Oral Presentation* (Team members; name of meeting; date of meeting; title of abstract; publication or availability details if applicable) | | | |
| 1. *Poster presentation* (Team members; name of meeting ; date of meeting; title of abstract; publication or availability details if applicable) | | | |
| 1. *Article* (Authors; title of article, title of journal, year of publication, volume number, page numbers) | | | |

*Thank you for your participation in the initiative and in the returning of this feedback.*

*The information contained herein is confidential*

*This feedback may be used for research purposes and return of the feedback is taken as consent.*

***To utilize this questionnaire please request permission from Dr. Gabrielle McKee*** [***gmckee@tcd.ie***](mailto:gmckee@tcd.ie)
